# Surgical site infections after emergency hernia repair: substudy from the Management of Acutely Symptomatic Hernia (MASH) study

**DOI:** 10.1093/bjsopen/zrac155

**Published:** 2023-01-12

**Authors:** Victoria K Proctor, Olivia M O’Connor, Flora A Burns, Susie Green, Adele E Sayers, Deborah J Hawkins, Neil J Smart, Matthew J Lee, MASH Collaborators, D Hoban, A Kattakayam, R Lunevicius, G Madzamba, O Rutka, P Hopley, W Ibrahim, M Issa, D Nair, A Reddington, J Wilson, D Ashmore, R Clarke, A Daniels, L Harrison, S Hope, A Masri, M Albendary, H Harris, V Pegna, P Sains, NS Blencowe, E Kirkham, S Rozwadowski, E Martin, C McFaul, V Maxwell, J Morgan, T Wilson, A Belgaumkar, Z Elahi, J Ma, S Maher, P Narayan, B Oyewole, R Adair, J Cowley, B Dobbins, T Grey, A Jackson, M Junejo, M Peter, A Saha, A Findlay, G Kakaniaris, H O’Grady, A Wilkins, J Yau, T Bhuvanakrishna, O Jeepalaya, M Sinclair, M Dunstan, I Gerogiannis, T Pelly, J Vance-Daniel, L Gurowich, M Hollyman, L Merker, R Amjad, M Barghash, S Dalmia, L Morris, M Tarazi, S Daniels, N Husnoo, J Johnston, E Denis, C Hirst, J Lim, S Patil, J Sarveswaran, L Scott, I Bondoqa, N Carter, A Darbyshire, M Moon, S Toh, A Banerjea, Z Chia, J Curtis, J Jackman, T Kanani, C Lewis-Lloyd, A Morton, J Ng, M Shaw, K Topham, R Kelleher, S Moug, A Pollock, E Westwood, U Donigiewicz, GE Fowler, O Hartrick, A Kushairi, L Massey, L Park, N Rajaretnam, E Walker, S Gupta, L Smith, G Williams, M Boland, D Damaskos, M Drogouti, B Wilson, M Lim, V Miu, L Onos

**Affiliations:** Academic Directorate of General Surgery, Sheffield Teaching Hospitals NHS Foundation Trust, Sheffield, UK; Academic Directorate of General Surgery, Sheffield Teaching Hospitals NHS Foundation Trust, Sheffield, UK; Academic Directorate of General Surgery, Sheffield Teaching Hospitals NHS Foundation Trust, Sheffield, UK; Department of General Surgery, York Teaching Hospitals, York, UK; Academic Directorate of General Surgery, Sheffield Teaching Hospitals NHS Foundation Trust, Sheffield, UK; Academic Directorate of General Surgery, Sheffield Teaching Hospitals NHS Foundation Trust, Sheffield, UK; Department of General Surgery, Royal Devon and Exeter Hospital, Exeter, UK; Academic Directorate of General Surgery, Sheffield Teaching Hospitals NHS Foundation Trust, Sheffield, UK; Department of Oncology and Metabolism, University of Sheffield, Sheffield, UK

## Abstract

**Introduction:**

Acutely symptomatic abdominal wall and groin hernias (ASH) are a common acute surgical presentation. There are limited data to guide decisions related to surgical repair technique and use of antibiotics, which can be driven by increased risk of surgical site infection (SSI) in this group. This study aims to report rates of SSI following ASH repair and explore the use of patient-reported outcome measure reporting in this setting.

**Methods:**

An 18-week, UK-based, multicentre prospective cohort study (NCT04197271) recruited adults with ASH. This study reports operatively managed patients. Data on patient characteristics, inpatient management, quality of life, complications, and wound healing (Bluebelle score) were collected. Descriptive analyses were performed to estimate event rates of SSI and regression analysis explored the relationship between Bluebelle scores and SSI. The 30 and 90-day follow-up visits assessed complications and quality of life.

**Results:**

The MASH study recruited 273 patients, of whom 218 were eligible for this study, 87.2 per cent who underwent open repair. Mesh was used in 123 patients (50.8 per cent). Pre- and postoperative antibiotics were given in 163 (67.4 per cent) and 28 (11.5 per cent) patients respectively. There were 26 reported SSIs (11.9 per cent). Increased BMI, incisional, femoral, and umbilical hernia were associated with higher rates of SSI (*P* = 0.006). In 238 patients, there was a difference in healthy utility values at 90 days between patients with and without SSI (*P* = 0.025). Also, when analysing 191 patients with Bluebelle scores, those who developed an SSI had higher Bluebelle values (*P* < 0.001).

**Conclusion:**

SSI is frequent in repair of acutely symptomatic hernia and correlates with BMI and site of hernia.

## Teaser

This study reports surgical site infection rates after emergency hernia repair. It also captures data on quality of life, and the behaviour of a wound health patient-reported outcome measure in this cohort.

Registration number: NCT04197271 (http://www.clinicaltrials.gov).

## Introduction

Acutely symptomatic hernias (ASH) of the abdominal wall and groin are a common acute surgical presentation with a quarter of all hernia repairs being performed as an emergency^1^. In the emergency setting, the patient is more likely to present with a complicated hernia, meaning that the contents may be incarcerated, obstructed, or strangulated. Where obstructed or ischaemic tissue is involved, the risk of surgical site infection (SSI) is often judged to be higher. Decisions related to repair technique, specifically the use of mesh, can be driven by contamination and perceived SSI risk, as mesh infection can be a difficult and costly problem to treat, with prolonged hospital stays and further surgery required.

There is limited evidence to guide the use of mesh in emergency hernia repair where the risk of SSI is often felt to be higher than in elective surgery. Most guidelines suggest that the approach should be ‘tailored’ for individual patients^2–4^. The use of prophylactic antibiotics to reduce the risk of SSI in emergency hernia repair is a further area of uncertainty, with only weak evidence to support the use of antibiotics for 48–72 h in clean-contaminated or contaminated cases^4^.

After emergency surgery, many patients do not undergo routine surgical follow-up and often seek advice and support from other healthcare sources, such as their general practitioner, with postoperative problems. This means that the true incidence of SSI after surgery for ASH may be higher than previously thought.

Therefore, the aims of this study are to report rates of SSI following ASH repair and to explore factors associated with SSI, using data from the completed Management of Acutely Symptomatic Hernia (MASH) study^5^. MASH was designed as a UK multicentre prospective cohort study and aimed to assess outcomes and management strategies of emergency hernia surgery, with a high-level description of patients. The present substudy focused on the rates of SSI, associated management strategies, and SSI-related patient-reported outcomes (PROMs) in the surgery group.

## Methods

The MASH study was a UK-based prospective cohort study (NCT04197271). It was conducted over a 20-week interval, in 23 UK-based centres. Ethical approval was secured from the West of Scotland NHS Research Ethics Committee (19/WS/0182).

### Eligibility criteria

Patients eligible for participation in MASH were adults aged >16 years, presenting to hospital with an acutely symptomatic femoral, inguinal, umbilical/paraumbilical, epigastric, incisional, or perineal hernia. Patients had to be able to provide consent to participate. Those unable to provide consent and pregnant women were considered not eligible.

### Recruitment

Participants were screened for eligibility and recruited by local research teams. Potential participants were provided with written information and allowed a short interval (at least an hour) between presentation of information and consent. Participants were permitted to withdraw at any time without giving a reason.

### Data collection

Following consent, demographic information, hernia details, and data relating to initial investigation and assessment of patients were captured. Details on management strategy (early surgery, less than 24 h after admission), delayed surgery (a ‘soon’ elective list), palliation, or conservative management. If managed conservatively, reasons for this were captured. Data were captured on aspects of surgical management, including operating approach, anaesthesia modality, use of antibiotics, and material used for repair. Antibiotic type was not recorded as these are delivered according to local guidance in UK hospitals, respecting local populations and microbial sensitivities. Prophylactic antibiotics were those given before knife to skin, and postoperative antibiotics were those given for any interval after surgery.

### Outcomes of interest

Outcome data collected included the occurrence of SSI, hospital duration of stay, Bluebelle wound health questionnaire (WHQ) score, EuroQol-5D-5L, and in hospital complications. Complications were accepted as having occurred if documented by the responsible clinical team. These included pneumonia, urinary tract infection, and delirium.

SSI was based on assessment by the responsible surgical team. This was assessed at discharge, 30 days, and 90 days after surgery. This was defined in terms of US Centers for Disease Control and Prevention (CDC) criteria as a diagnosis made by a clinician and affecting the superficial surgical wound site; however, the study allowed SSI to be diagnosed up to 90 days not 30 days^6^.

Participants completed the Bluebelle WHQ via telephone at 30 and 90 days after surgery (±7 days)^7^. The Bluebelle WHQ is a tool designed for patient self-reporting of SSI. It was designed and developed across a range of general surgical procedures, including acute hernia^7^. It includes questions about symptoms including redness, discharge, fever, and wound dehiscence and it has shown good sensitivity and specificity in the detection of SSI^7^. It generates a score between 0–40, with a value of eight or higher suggesting occurrence of SSI. Participants also completed a generic quality of life measure (EQ-5D-5L) at admission, 30 days and 90 days after discharge (±7 days). Data were entered onto a central Redcap database^8^, hosted at the University of Sheffield, UK.

### Statistics

For the purpose of this study, only patients who had undergone surgical treatment of their hernia were eligible for inclusion. This data set was used to estimate event rates of SSI using descriptive statistics (Fisher’s exact test, chi-squared, and Wilcoxon rank sum) as appropriate using R^9^ and the Tidyverse package^10^. Health utility was calculated by cross-walking EQ-5D-5L to UK reference values using the EuroQol crosswalk calculator^11^. The data set was then interrogated to identify those participants with at least one Bluebelle data set completed. Bluebelle scores were calculated as recommended by the development team^7^. The higher of the two Bluebelle scores was used in the analysis. Sensitivity, specificity, and negative and positive predictive values were calculated for different diagnostic cut-off scores. Finalfit^12^ was used to explore the association between Bluebelle score and SSI presence by logistic regression using Bluebelle as explanatory factor for SSI presence, and linear regression using SSI presence as an explanatory factor in Bluebelle score to demonstrate presumed co-linearity. A second linear regression model was developed to explore the relationship of clinical features with Bluebelle score.

## Results

### Participants and treatments

The MASH study recruited 273 patients, of whom 218 were eligible for inclusion. Of those included in the study, 168 (72.7 per cent) were male, the median BMI was 27.9 kg/m^2^ (interquartile range (i.q.r.) 24.8–33.0). Umbilical and inguinal were the most common hernia sites accounting for 93 cases each (38.4 per cent). The majority of hernias (216 (89.2 per cent)) were primary hernias that were not recurrent. Median white cell count and C-reactive protein (CRP) were 9.1 × 10^9^/l (i.q.r. 7.3–11.8) and 5.0 mg/l (i.q.r. 2.0–22.6) respectively. The most common hernia presentation was incarceration (119 (49.1 per cent)), and symptoms had been present for more than 24 h for 144 (59.5 per cent) patients (*Table 1*).

**Table 1:** Overall participants

Characteristic	Overall, *n* = 242	No SSI, *n* = 216	SSI, *n* = 26	P*
**Age (years), median (i.q.r.)**	65 (51, 75)	65 (50, 75)	64 (53, 69)	0.910
**Sex ratio (M:F)**	176:66	161:55	15:11	0.068
**BMI (kg/m^2^), median (i.q.r.)**	28 (25, 33)	27 (24, 32)	35 (29, 42)	<0.001
* *Unknown	14	13	1	
**Hernia site**				0.006
Epigastric	14 (5.8)	14 (6.5)	0 (0.0)	
Femoral	22 (9.1)	19 (8.8)	3 (11.5)	
Incisional	19 (7.9)	15 (6.9)	4 (15.4)	
Inguinal	93 (38.4)	90 (41.7)	3 (11.5)	
Perineal	1 (0.4)	1 (0.5)	0 (0.0)	
* *Umbilical	93 (38.4)	77 (35.6)	16 (61.5)	
**Clinical findings**				0.012
Symptomatic	66 (27.7)	65 (30.5)	1 (4.0)	
Incarcerated	119 (50.0)	102 (47.9)	17 (68.0)	
Obstructed	31 (13.0)	26 (12.2)	5 (20.0)	
Strangulated	22 (9.2)	20 (9.4)	2 (8.0)	
Unknown	4	3	1	
**Primary hernia**	216 (89.3)	194 (89.8)	22 (84.6)	0.498
**White cell count (10^9^/mm^3^), median (i.q.r.)**	9.1 (7.3, 11.8)	9.1 (7.2, 11.6)	9.4 (7.8, 12.9)	0.301
* *Unknown	4	4	0	
**C-reactive protein (mg/l), median (i.q.r.)**	5 (2, 23)	5 (2, 21)	8 (5, 31)	0.194
Unknown	23	22	1	

Values are *n* (%) unless otherwise indicated.*Wilcoxon rank sum test; Pearson's chi-squared test; Fisher's exact test. SSI, surgical site infection; i.q.r., interquartile range.

Hernias were typically repaired through an open approach (87.2 per cent). Mesh repair was performed for 123 (50.8 per cent) patients. Preoperative antibiotics were used in 163 patients (67.4 per cent), and 28 (11.5 per cent) received postoperative antibiotics (*Table 2*). Of the 57 patients who received no antibiotics, six (12 per cent) developed SSI. Of the 24 who received antibiotics before and after surgery, six (25 per cent) developed SSI; 116 received preoperative antibiotics only (10.3 per cent), and two received postoperative antibiotics only. Antibiotic data were missing for the remaining patients.

**Table 2:** Treatment characteristics

Characteristic	No SSI, *n* = 216	SSI, *n* = 26	P*
**Repair type**			0.292
Mesh	112 (57.1)	11 (45.8)	
Sutured	84 (42.9)	13 (54.2)	
Unknown	20	2	
**Operative approach**			0.212
Open	189 (94.5)	22 (88.0)	
Laparoscopic	8 (4.0)	2 (8.0)	
Laparoscopic converted to open	3 (1.5)	1 (4.0)	
Unknown	16	1	
**Duration of symptoms**			0.090
<24 h	83 (38.4)	14 (56.0)	
>24 h	133 (61.6)	11 (44.0)	
Unknown	0	1	
**Preoperative antibiotics given**	143 (73.0)	20 (76.9)	0.667
Unknown	20	0	
**Postoperative antibiotics given**	22 (12.3)	6 (25.0)	0.112
* *Unknown	37	2	

Values are *n* (%). *Fisher's exact test; Pearson's chi-squared test. SSI, surgical site infection.

### Characteristics and treatment effects associated with SSI

SSI was reported for 26 (11.9 per cent) patients. Characteristics associated with the development of SSI included increased BMI at 34.9 kg/m^2^ (i.q.r. 28.6–42.4) *versus* 27.4 kg/m^2^ (i.q.r. 24.5–32.0) (*P* < 0.001), *Table 1*. Age, sex, duration of symptoms, white cell count, and CRP were not associated with increased rates of SSI. Incisional, femoral, and umbilical, and femoral hernia had higher SSI rates than other sites (4 of 19 (21 per cent), 16 of 93 (17 per cent), and 3 of 22 (13 per cent) respectively (*P* = 0.006)). Hernias that were symptomatic only were associated with 2 per cent SSI rate, compared with 16 per cent obstructed, 14.2 per cent of incarcerated hernia, and 9 per cent strangulated hernia (*P* = 0.006). No statistical difference was observed in the rates of SSI according to the operating approach, duration of symptoms (more or less than 24 h), use of preoperative/postoperative antibiotics, or type of mesh used (*Tables 2* and *3*).

**Table 3:** Surgical site infection according to type of mesh used

Characteristic	No SSI, *n* = 112	SSI, *n* = 11	P*
**Mesh type**			0.140
Biological	3 (100)	0 (0)	
Synthetic absorbable	9 (75)	3 (25)	
Synthetic non-absorbable	100 (93)	8 (7.4)	

Values are *n* (%). *Fisher's exact test. SSI, surgical site infection.

### Health utility

**Fig. 1:**
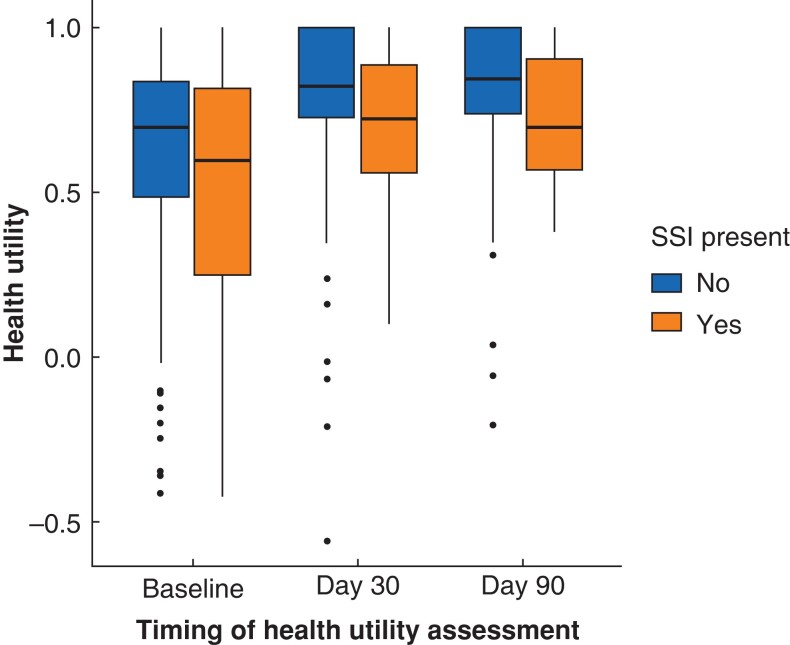
Health utility by presence of surgical site infection SSI, surgical site infection.

Health utility values were available for 238 patients at baseline, 185 at 30 days, and 162 at 90 days. There was no difference in health utility at baseline regardless of SSI development. At 30 days, there was a non-significant difference between groups with no-SSI or SSI having utility respectively of 0.82 (i.q.r. 0.73–1.00) compared with 0.72 (i.q.r. 0.55–0.88) (*P* = 0.52). A significant difference was noted between groups at 90 days, with the no-SSI group having a utility of 0.84 (i.q.r. 0.74–1.00) compared with the SSI group utility of 0.70 (i.q.r. 0.57–0.91) (*P* = 0.025) (*Fig. 1*).

### Bluebelle scores

Bluebelle scores were available for 191 patients, of whom 24 (12.5 per cent) had a documented SSI. The median score overall was 9 (i.q.r. 8–10). The group who developed SSI had significantly higher Bluebelle scores (median 12 (i.q.r. 9–23) *versus* median 9 (i.q.r. 8–10) respectively, *P* < 0.001). Where the diagnostic cut-off of a score of eight or more was applied, 135 of 167 (80.8 per cent) in the no-SSI group would have been incorrectly triaged, and 20 of 24 (83 per cent) of the SSI group would have been correctly triaged. If a cut-off of 10 was used, 47 of 167 (28.1 per cent) of the no-SSI group would have been incorrectly triaged and 16 of 24 (66 per cent) of the SSI group would have been correctly triaged. A cut-off score of eight provided a negative predictive value for SSI in 88.9 per cent cases, *versus* 85.5 per cent for a cut-off of 10. Sensitivity and specificity are presented in *Table 4*.

**Table 4:** Performance of different Bluebelle score cut-offs

	Cut-off 8	Cut-off 10
True positive	20	16
False positive	135	47
True negative	32	120
False negative	40	8
Sensitivity (%, 95% c.i.)	83.3 (62.6,95.2)	66.7 (44.6,84.4)
Specificity (%, 95% c.i.)	19.1 (13.5,25.9)	28.1 (21.4,35.6)
Positive predictive value (%, 95% c.i.)	12.8 (10.8,15.2)	11.7 (8.9,15.1)
Negative predictive value (%, 95% c.i.)	88.9 (75.7,95.4)	85.5 (76.1,91.6)

Values are *n* unless otherwise indicated.

**Fig. 2:**
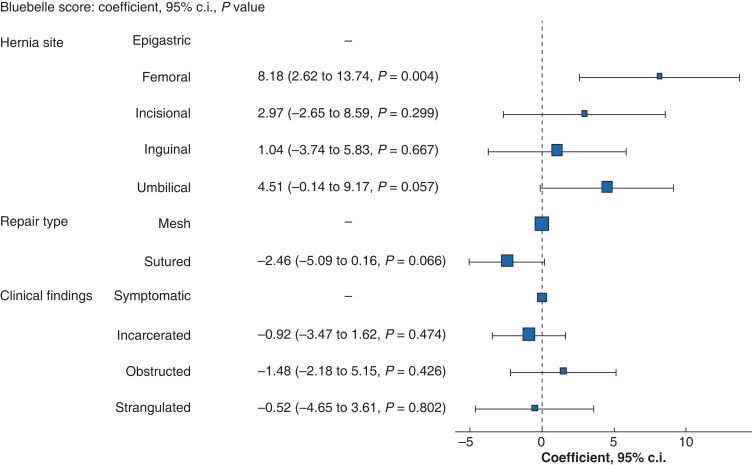
Coefficient plot demonstrating relationship between characteristics and Bluebelle score

Logistic regression confirmed the association between Bluebelle and SSI presence, with an OR of 1.07 (95 per cent c.i. 1.02 to 1.13, *P* = 0.003) for each point increase in Bluebelle score. The c-statistic for this model was 0.716, showing moderate performance. Linear regression showed the presence of an SSI had a positive coefficient related to Bluebelle score of 5.30 (95 per cent c.i. 2.21 to 8.38, *P* = 0.001). A second linear regression model demonstrated that the presence of a femoral hernia was associated with increased Bluebelle score (8.18 (2.62 to 13.74), *P* = 0.004). A borderline positive correlation was seen between an umbilical hernia and Bluebelle score (4.51 (−0.14 to 9.17), *P* = 0.057). Sutured repair showed a negative direction of association, but this was not significant (−2.46 (−5.09 to 0.16), *P* = 0.066). None of the clinical findings showed an association with score (*Fig. 2*). When the same characteristics were modelled against the presence of SSI, none of the variables demonstrated significance (*Table S1*).

## Discussion

This study has shown that SSI is common in repair of ASH, affecting around one in 10 cases. SSI was most commonly seen in those with increased BMI and incisional hernia. Previous work has demonstrated the association of these factors with subsequent SSI^13,14^. While these factors, along with diabetes mellitus and smoking status are preferably optimized in the elective setting, there is no meaningful opportunity to mitigate these risks in the acute setting. This means that strategies to reduce SSI will be more reliant on technical aspects of management.

In this cohort, open repair was typically performed. This has also been associated with higher SSI rates in the elective setting^15^. Several published cohorts demonstrate the feasibility and safety of incarcerated laparoscopic repair of groin, ventral, and incisional hernia^16–18^. While laparoscopic hernia repair has a relatively steep learning curve, it is interesting that this core area of emergency surgery has not mirrored the uptake of laparoscopy seen in other emergency abdominal conditions in the UK setting^19^. This likely reflects low rates of elective repair in the UK (approximately 17.1 per cent for primary unilateral inguinal hernia)^20^.

The choice of repair material (suture *versus* mesh) did not seem to be associated with the occurrence of SSI, in keeping with other published studies^21^. This may reflect surgical expertise recognizing a field that is inhospitable to mesh using it where necessary, or it may inform us of an overly cautious approach to mesh use in the emergency setting. While data on the types of suture and mesh used were collected, these have not been presented as the small numbers of SSI occurrence would lead to excessive granularity of data.

There is no strong evidence for the use of antibiotic prophylaxis in elective repair of a ‘clean’ hernia^22^. Guidance is lacking on the use of prophylactic and postoperative antibiotic use in the emergency setting. The role of antibiotics here is not simply prophylaxis of skin flora implantation, it may also include coverage of translocated bacteria from ischaemic bowel. Treatment may also be required after surgery to further reduce this risk. The World Society of Emergency Surgery offers weak recommendations on the use of antibiotics for 48–72 h after a clean-contaminated or contaminated case^4^. This uncertainty was reflected here with only a small number of patients receiving pre- or postoperative antibiotic therapy. Clearly, more robust evidence is required. The authors acknowledge that the failure to capture specific type of antibiotic use means that useful data might be missing; however, with the number of patients receiving antibiotics and SSI rates recorded, these data are likely to have been too granular to be of use.

This study provides new data on quality of life and use of a wound PROM in acute hernia. In this study, there was a non-significant difference in quality of life at 30 days, although this approached significance. This became significantly different by 90 days, with a difference of 0.14. Despite searching, it has not been possible to identify a minimum clinically important difference for health utility after hernia repair, therefore it was not possible to say whether this value is significant enough to have a clinically detectable output.

A novel feature of this study is the capture of Bluebelle WHQ data for 45 per cent of participants. The validation of this tool did include 61 patients following groin hernia repair^7^. The findings of the study show that a Bluebelle score higher than eight demonstrates good performance in the detection of SSI. The model of factors suggests that femoral hernia and use of mesh in repair are associated with increased scores. This means that the scores may not be artefactual, but may reflect hernia repairs in a sensitive area (femoral region), associated with increased BMI (umbilical), and problems related to mesh sensation. With these caveats, the Bluebelle score seems to function well in this cohort. When comparing the relationship of factors against Bluebelle score and SSI presence, the Bluebelle score seemed to show associations with clinical characteristics, whereas SSI did not. Some of these may not have reached significance due to the overall sample size, but may be worth exploring in future work.

The original MASH study was not designed with SSI as a primary endpoint and was conducted during the COVID-19 pandemic. This means there are some limitations around assessment of wounds, patient selection, and follow-up. Specifically, patients were not asked to return for assessment of their wounds due to risks of unnecessary hospital attendance. Participants may have attended alternative healthcare settings for treatment of SSI, meaning the data presented here may be an underestimate of the true event rate. There was also moderate attrition in the Bluebelle group and these missing data may affect findings. As the study was conducted during the COVID-19 pandemic where increasingly stringent hygiene and cleanliness protocols were used, it is possible that the rate recorded here is lower than might otherwise have been expected.

This study used a prospective multicentre approach to report the current rates of SSI in ASH. It demonstrated variation in aspects of care that might be used to reduce SSI in this setting. The study has also incorporated a PROM in the form of Bluebelle and provided insights that will support its use in future studies. Clinicians and policy makers should ensure they have accurate data on SSI incidence and outcomes in their ASH practice, especially as this has implications on recurrence^23^. This monitoring could be supported by integration of Bluebelle into routine clinical care. Consideration should be given to ensuring the use of best practice in reducing SSI. The study highlights areas of variation that may benefit from exploration in a randomized trial, including repair strategies and perioperative antibiotic use.

## Collaborators

### MASH Collaborators

D. Hoban, A. Kattakayam, R. Lunevicius, G. Madzamba, and O. Rutka (Aintree University Hospital, Liverpool, UK); P. Hopley, W. Ibrahim, M. Issa, D. Nair, A. Reddington, and J. Wilson (Arrowe Park Hospital, Birkenhead, UK); D. Ashmore, R. Clarke, A. Daniels, L. Harrison, S. Hope, and A. Masri (Barnsley Hospital, Barnsley, UK); M. Albendary, H. Harris, V. Pegna, and P. Sains (Brighton and Sussex University Hospitals, Brighton, UK); N.S. Blencowe, E. Kirkham, and S. Rozwadowski (Bristol Royal Infirmary, Bristol, UK); E. Martin and C. McFaul (Countess of Chester Hospital, Chester, UK); V. Maxwell, J. Morgan, and T. Wilson (Doncaster Royal Infirmary, Doncaster, UK); A. Belgaumkar, Z. Elahi, J. Ma, S. Maher, P. Narayan, and B. Oyewole (East Surrey Hospital, Redhill, UK); R. Adair, J. Cowley, B. Dobbins, T. Grey, A. Jackson, M. Junejo, M. Peter, and A. Saha (Huddersfield Royal Infirmary, Huddersfield, UK); A. Findlay, G. Kakaniaris, H. O’Grady, A. Wilkins, and J. Yau (Hull University Hospitals, Hull, UK); T. Bhuvanakrishna, O. Jeepalaya, and M. Sinclair (Ipswich Hospital, Ipswich, UK); M. Dunstan, I. Gerogiannis, T. Pelly, and J. Vance-Daniel (Kingston Hospital, Kingston upon Thames, UK); L. Gurowich, M. Hollyman, and L. Merker (Musgrove Park Hospital, Taunton, UK); R. Amjad, M. Barghash, S. Dalmia, L. Morris, and M. Tarazi (North Manchester General Hospital, Manchester, UK); S. Daniels, N. Husnoo, and J. Johnston (Northern General Hospital, Sheffield, UK); E. Denis, C. Hirst, J. Lim, S. Patil, J. Sarveswaran, and L. Scott (Pinderfields Hospital, Wakefield, UK); I. Bondoqa, N. Carter, A. Darbyshire, M. Moon, and S. Toh (Queen Alexandra Hospital, Portsmouth, UK); A. Banerjea, Z. Chia, J. Curtis, J. Jackman, T. Kanani, C. Lewis-Lloyd, A. Morton, J. Ng, M. Shaw, and K. Topham (Queen’s Medical Centre, Nottingham, UK); R. Kelleher, S. Moug, A. Pollock, and E. Westwood (Royal Alexandra Hospital, Paisley, UK); U. Donigiewicz, G.E. Fowler, O. Hartrick, A. Kushairi, L. Massey, L. Park, N. Rajaretnam, and E. Walker (Royal Devon and Exeter Hospital, Exeter, UK); S. Gupta, L. Smith, and G. Williams (Royal Gwent Hospital, Newport, UK); M. Boland, D Damaskos, M. Drogouti, and B. Wilson (Royal Infirmary of Edinburgh, Edinburgh, UK); and M. Lim, V. Miu, and L. Onos (York Hospital, York, UK).

## Supplementary Material

zrac155_Supplementary_Data

## Data Availability

The data sets generated during and/or analysed during this study may be available from the corresponding author on reasonable request.
