# Supplementary material for: Surgical site infections after emergency hernia repair: substudy from the Management of Acutely Symptomatic Hernia (MASH) study
Source: BJS Open. 2023 Jan 12;7(1):zrac155. doi: 10.1093/bjsopen/zrac155 (PMC9835494; doi:10.1093/bjsopen/zrac155)
Supplement: zrac155_Supplementary_Data [file zrac155_supplementary_data.docx]

**Surgical site infections following emergency hernia repair: sub-study from the Management of Acutely Symptomatic Hernia (MASH) study.**

**VK Proctor^1^, OM O’Connor^1^, FA Burns^1^, S Green^2,^ AE Sayers^1^, DJ Hawkins^1^ NJ Smart^3^, MJ Lee^1,4^ on behalf of the MASH Collaborators**

Affiliations

1. Academic Directorate of General Surgery, Sheffield Teaching Hospitals NHS Foundation Trust, Sheffield, UK
2. Department of General Surgery, York Teaching Hospitals, York, UK,
3. Department of General Surgery, Royal Devon and Exeter Hospital, Exeter, UK
4. Department of Oncology and Metabolism, University of Sheffield, Sheffield, UK

Corresponding author details

Matthew Lee, FU32, Department of Oncology and Metabolism, The Medical School, University of Sheffield, S10 2RX

[m.j.lee@sheffield.ac.uk](mailto:m.j.lee@sheffield.ac.uk), @wannabehawkeye ORCID: https://orcid.org/0000-0001-9971-1635

**Supplementary Materials - Index**

| **Supplementary Figures and tables** |  |
| --- | --- |
| Table S1. | *pag. 2* |
|  |  |
|  |  |

**Table S1.**

| Dependent: SSI present |  | No | Yes | OR (univariable) | OR (multivariable) |
| --- | --- | --- | --- | --- | --- |
| Hernia Site | Epigastric | 11 (100.0) |  | - | - |
|  | Femoral | 16 (88.9) | 2 (11.1) | 5318101.69 (0.00-NA, p=0.990) | 4553466.18 (0.00-5.12x10^29^, p=0.989) |
|  | Incisional | 13 (76.5) | 4 (23.5) | 13090711.85 (0.00-NA, p=0.989) | 9534496.51 (0.00-NA, p=0.989) |
|  | Inguinal | 72 (96.0) | 3 (4.0) | 1772700.56 (0.00-NA, p=0.990) | 1701779.09 (0.00-NA, p=0.990) |
|  | Umbilical | 55 (78.6) | 15 (21.4) | 11603130.95 (0.00-NA, p=0.989) | 10730984.06 (0.00-5.21x10^29^, p=0.989) |
| Repair Type | Mesh | 99 (90.0) | 11 (10.0) | - | - |
|  | Sutured | 68 (84.0) | 13 (16.0) | 1.72 (0.73-4.14, p=0.216) | 0.76 (0.27-2.21, p=0.614) |
| Clinical Findings | Symptomatic | 48 (98.0) | 1 (2.0) | - | - |
|  | Incarcerated | 81 (84.4) | 15 (15.6) | 8.89 (1.72-163.25, p=0.037) | 7.31 (1.35-136.14, p=0.061) |
|  | Obstructed | 20 (80.0) | 5 (20.0) | 12.00 (1.79-237.80, p=0.027) | 8.56 (1.17-175.80, p=0.064) |
|  | Strangulated | 15 (88.2) | 2 (11.8) | 6.40 (0.57-143.54, p=0.141) | 3.84 (0.33-88.34, p=0.294) |

SSI does not have same association with clinical features as Bluebelle.
